# Supplementary material for: Comparison of Stir Bar Sorptive Extraction and Solid Phase Microextraction of Volatile and Semi-Volatile Metabolite Profile of Staphylococcus aureus
Source: Molecules. 2019 Dec 23;25(1):55. doi: 10.3390/molecules25010055 (PMC6982899; doi:10.3390/molecules25010055)
Supplement: Supplementary file 1 [file molecules-25-00055-s001.pdf]

<u>**Supplementary Materials**</u>

Comparison of stir bar sorptive extraction and solid phase microextraction of volatile and semi-volatile metabolite profile of *Staphylococcus aureus*

Kevin Berrou[1], Catherine Dunyach-Remy[2,3], Jean-Philippe Lavigne[2,3], Benoit Roig[1], Axelle Cadiere[1]

[1] Univ. Nimes, EA7352 CHROME, Rue du Dr G. Salan, 30021, Nimes Cedex 1, France

[2] Institut National de la Santé et de la Recherche Médicale, U1047, Université Montpellier, UFR de Médecine, 30908 Nîmes, France.

[3] Department of Microbiology, CHU Nîmes, Univ Montpellier, 30029 Nîmes, France.

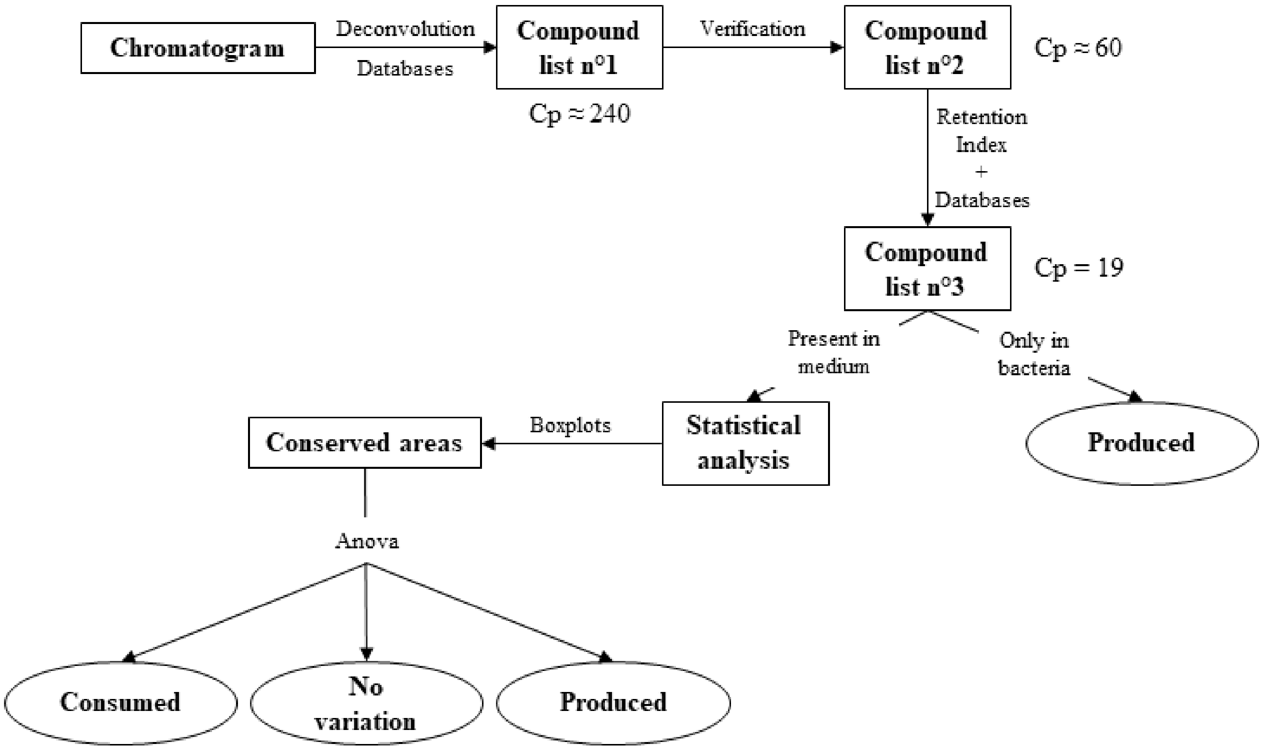

Supplementary Figure S1. Graphical summary of the methodology treatment of GC data. The number of compounds retained at each step is indicated by the abbreviation "Cp".

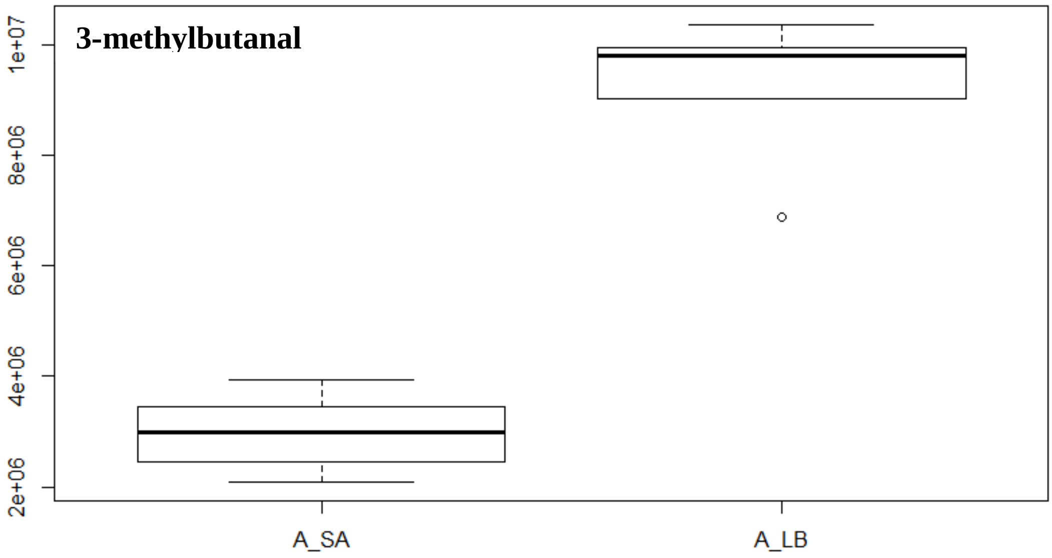

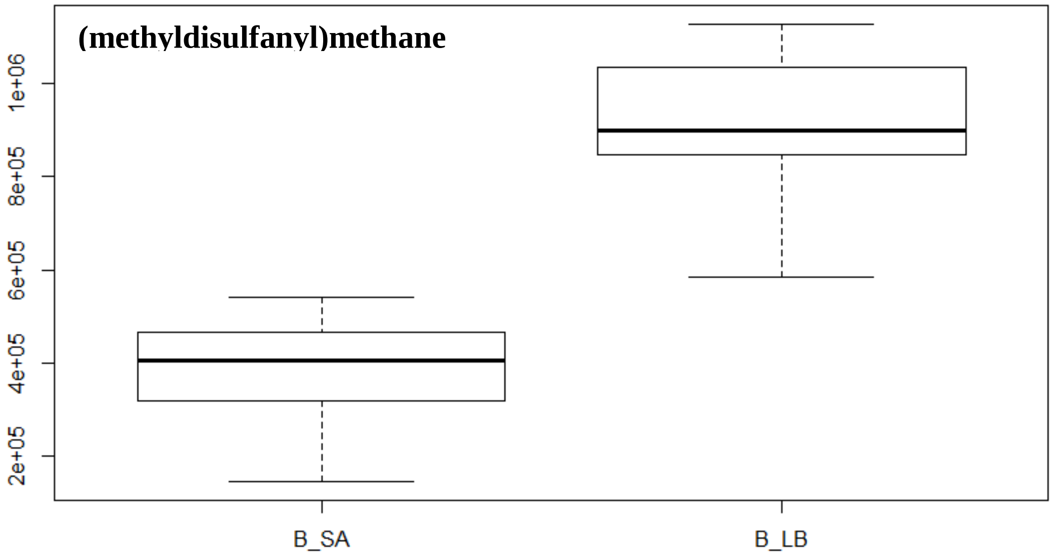

Supplementary Figure S2. Processing of SPME data by boxplot, carried out using RStudio software. Only the areas common to both conditions (bacterial culture (**SA**) and control (**LB**)) were analysed. Outliers are represented by points outside the boxplots, which will then be deleted from the area comparison analyses.

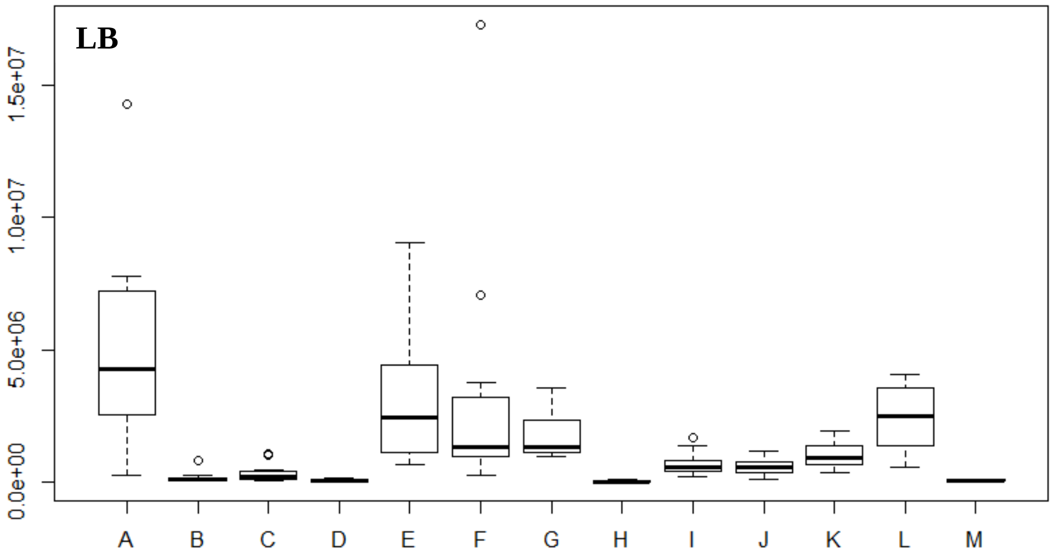

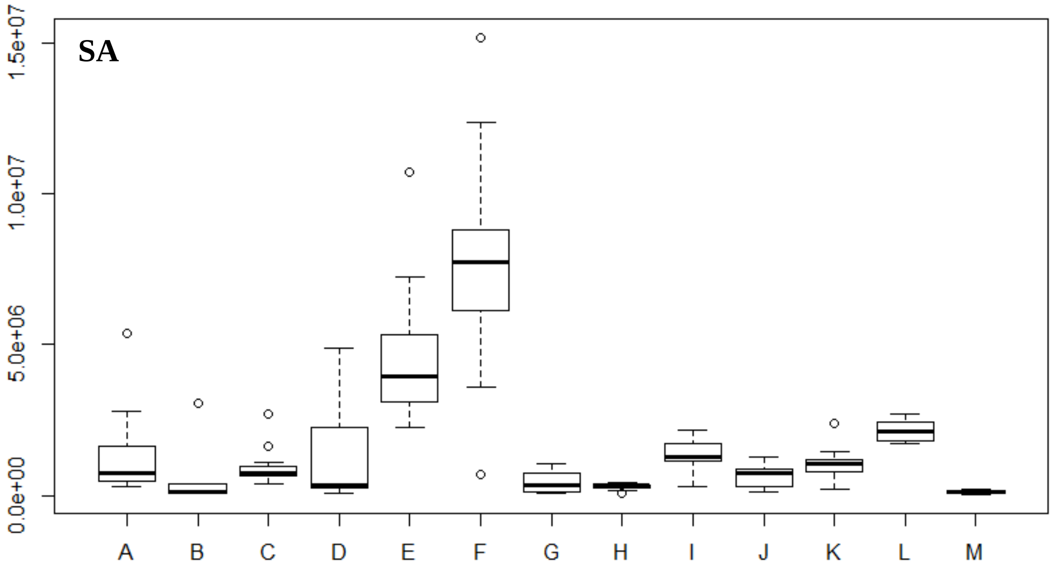

Supplementary Figure S3. Processing of SBSE data by boxplot, carried out using RStudio software. Only the areas common to both conditions (bacterial culture (**SA**) and control (**LB**)) were analysed. Outliers are represented by points outside the boxplots, which will then be deleted from the area comparison analyses. **A:** Acetaldehyde, **B:** Propan-2-one, **C:** Ethanol, **D:** (methyltrisulfanyl)methane, **E:** Acetic acid, **F:** Formic acid, **G:** Benzaldehyde, **H:** 2-hydroxybenzaldehyde, **I:** 1,3,5,7-Tetraazatricyclo[3.3.1.1^{3,7}]decane, **J:** 4-methylquinoline, **K:** Isoquinoline-1-carbonitrile, **L:** Quinoline-4-carbaldehyde, **M:** 1H-indole

Supplementary Table S1. Mean area of each metabolite identified after SBSE/HSSE extraction ([a]), SBSE/HSSE and SPME extraction ([b]) and SPME extraction ([c]). The results of the Anova statistical test performed on the 12 replicates are also presented.

Volatile metabolite	Mean area		p-value
	Bacterial culture	Control	
Acetaldehyde[a]	973104	3967090	3.12×10^{-3}
Propan-2-one[b]	178196	106356	4.80×10^{-5}
3-methylbutanal[c]	2961625	9195493	6.38×10^{-5}
Ethanol[a]	709758	207125	5.32×10^{-6}
(methyldisulfanyl)methane[c]	380742	909914	2.92×10^{-4}
1-methyl-1-propylhydrazine[a]	523915		
(methyltrisulfanyl)methane[a]	1354925	85454	3.38×10^{-2}
3-ethyl-2,5-dimethylpyrazine[a]	116597		
Acetic acid[a]	4061791	2173260	7.54×10^{-3}
Formic acid[a]	7584816	1548316	4.89×10^{-6}
Benzaldehyde[a]	445091	1779590	1.97×10^{-4}
3-methylbutanoic acid[b]	6220636		
2-hydroxybenzaldehyde[a]	323812	46307	2.41×10^{-7}
Acetamide[a]	219024		
1,3,5,7-Tetraazatricyclo[3.3.1.1^{3,7}]decane[a]	1306459	697443	1.88×10^{-3}
4-methylquinoline[a]	648680	592208	6.90×10^{-1}
Isoquinoline-1-carbonitrile[a]	916346	1019306	6.12×10^{-1}
Quinoline-4-carbaldehyde[a]	2133235	2399728	5.37×10^{-1}
1H-indole[a]	114534	72064	2.76×10^{-2}
